# The Coordination of Ethylene and Other Hormones in Primary Root Development

**DOI:** 10.3389/fpls.2019.00874

**Published:** 2019-07-10

**Authors:** Hua Qin, Lina He, Rongfeng Huang

**Affiliations:** ^1^Biotechnology Research Institute, Chinese Academy of Agricultural Sciences, Beijing, China; ^2^National Key Facility of Crop Gene Resources and Genetic Improvement, Beijing, China; ^3^College of Biological Sciences and Biotechnology, Beijing Forestry University, Beijing, China

**Keywords:** ethylene, hormones, primary root, elongation, crosstalk

## Abstract

The primary root is the basic component of root systems, initiates during embryogenesis and develops shortly after germination, and plays a key role in early seedling growth and survival. The phytohormone ethylene shows significant inhibition of the growth of primary roots. Recent findings have revealed that the inhibition of ethylene in primary root elongation is mediated *via* interactions with phytohormones, such as auxin, abscisic acid, gibberellin, cytokinins, jasmonic acid, and brassinosteroids. Considering that *Arabidopsis* and rice are the model plants of dicots and monocots, as well as the fact that hormonal crosstalk in primary root growth has been extensively investigated in *Arabidopsis* and rice, a better understanding of the mechanisms in *Arabidopsis* and rice will increase potential applications in other species. Therefore, we focus our interest on the emerging studies in the research of ethylene and hormone crosstalk in primary root development in *Arabidopsis* and rice.

## Introduction

As an underground organ of plants, the root system plays a vital role in the absorption and translocation of water and nutrients. The root system generally consists of two principal root types: the primary root and secondary roots. Primary root is formed embryonically. It is the basic component of the root system that absorbs mineral nutrients and provides mechanical support for shoot growth in young seedlings (Zheng et al., 2016). The growth of primary roots is maintained by two basal developmental processes: cell proliferation in the root apical meristem (RAM) and cell elongation in the elongation zone. Phytohormones are central regulators of plant root growth and development. Multiple phytohormones, including ethylene, auxin, abscisic acid (ABA), gibberellin (GA), cytokinin (CK), jasmonic acid (JA), strigolactone (SL), and brassinosteroid (BR), have been shown to play vital roles in the regulation of primary root growth (Li et al., 2015; Pacifici et al., 2015; Hu et al., 2017; Qin and Huang, 2018).

**Figure 1:**
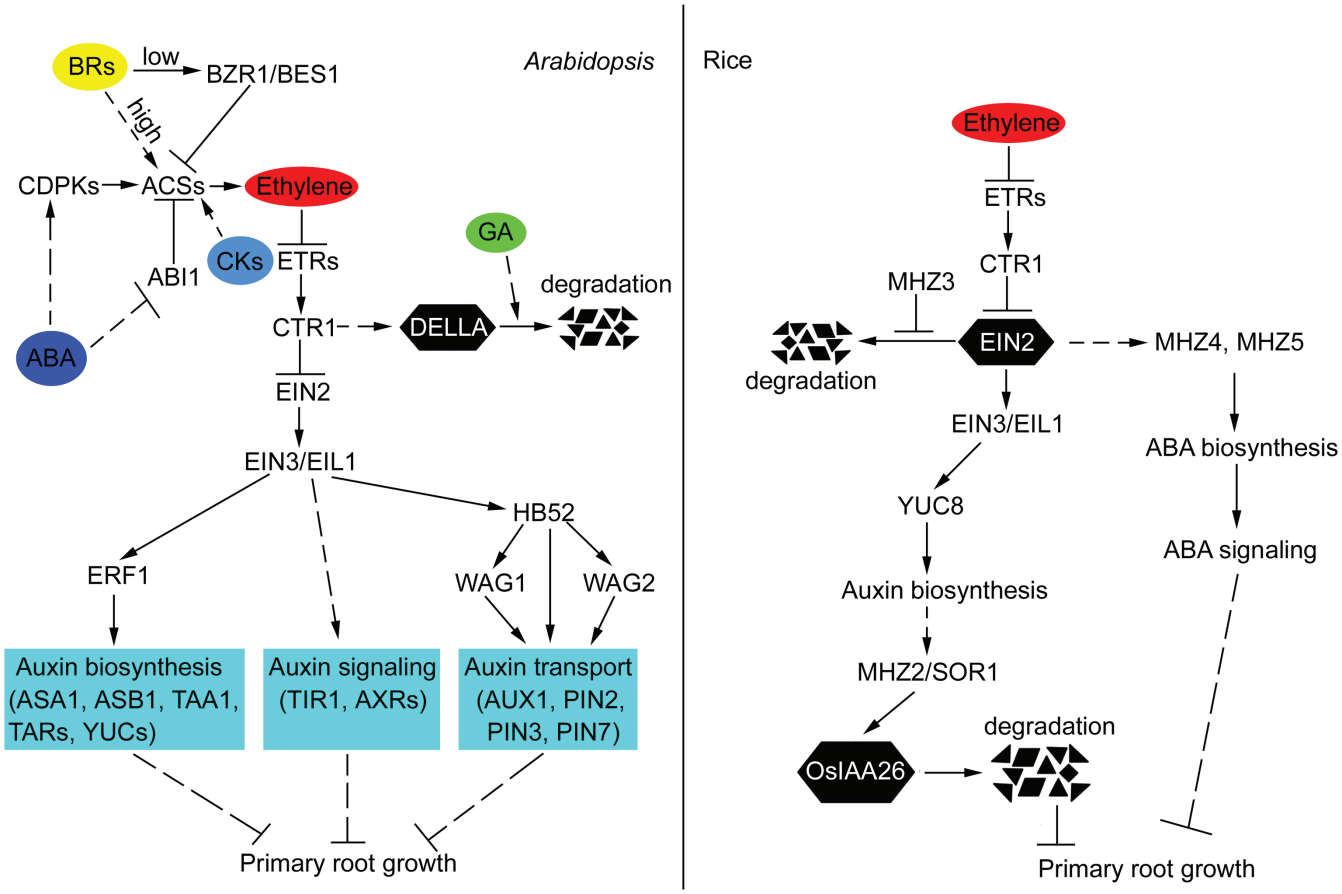
Crosstalk of ethylene and other hormones in the primary root growth of *Arabidopsis* and rice. In *Arabidopsis*, ethylene inhibits primary root growth by regulating auxin biosynthesis, transport, and signaling. EIN3/EIL1, ERF1, and HB52 function as crosstalk nodes between ethylene and auxin in this process. ABA promotes ethylene biosynthesis by affecting the posttranscriptional regulation of ACS. GA and ethylene antagonistically regulate the stability of DELLA proteins, which act as growth repressors. CKs induce ethylene biosynthesis by stabilizing ACS stability. Low concentrations of BRs inhibit ethylene biosynthesis by activating BZR1 and BES1 to repress the expression of *ACSs*. High levels of BRs induce ethylene biosynthesis through increasing the stability of ACSs. In rice, ethylene restricts primary root growth by increasing auxin and ABA biosynthesis. Auxin accumulation promotes SOR1-mediated degradation of OsIAA26, thus repressing normal root growth. MHZ3 stabilizes OsEIN2 to facilitate ethylene signal transduction. The solid lines indicate direct interactions, and the dashed lines indicate indirect interactions. The arrows indicate stimulatory effects, whereas the T sharp symbol indicates inhibitory effects.

Ethylene is a gaseous plant hormone that is synthesized from S-adenosylmethionine (SAM), which is converted to 1-aminocyclopropane-1-carboxylate (ACC) by ACC synthase (ACS). ACC is then converted to ethylene by ACC oxidase (ACO; Yang and Hoffman, 1984). Ethylene is perceived by a family of membrane-bound receptors (Hua et al., 1998; Hua and Meyerowitz, 1998). These receptors then inhibit the function of the Ser/Thr kinase CONSTITUTIVE TRIPLE RESPONSE1 (CTR1; Kieber et al., 1993), leading to the dephosphorylation and C-terminal cleavage of ETHYLENE INSENSITIVE2 (EIN2; Alonso et al., 1999; Ju et al., 2012; Qiao et al., 2012; Wen et al., 2012; Salehin and Estelle, 2015). The split EIN2 C-terminus translocates into the nucleus where it enables activation of the master transcriptional regulators EIN3 and EIN3-LIKE1 (EIL1), resulting in the induction of ethylene-triggered transcriptional response (Chao et al., 1997). In *Arabidopsis*, EIN2 is regulated by proteasomal degradation through EIN2 TARGETING PROTEIN1/2 (ETP1/2), whereas Maohuzi3 (MHZ3) protects OsEIN2 from proteasome-mediated degradation in rice (Figure 1; Qiao et al., 2009; Ma et al., 2017). Mutants with enhanced ethylene biosynthesis or signaling exhibit short primary roots (Kieber et al., 1993; Vogel et al., 1998; Woeste et al., 1999). By contrast, loss-of-function mutations of ethylene signaling components such as *ein2*, *ein3*, and *eil1* or treatment of seedlings with ethylene inhibitors such as the biosynthesis inhibitors 2-aminoethoxyvinylglycin (AVG; Ruzicka et al., 2007) and pyrazinamide (PZA; Sun et al., 2017), as well as the perception inhibitor silver nitrate (AgNO_3_; Ruzicka et al., 2007) and 1-methylcyclopropene (1-MCP; Serek et al., 1995) lead to increased primary root length and reduced ethylene response (Chao et al., 1997; Alonso et al., 1999; Ma et al., 2013; Yang et al., 2015).

An increasing number of investigations have revealed that ethylene affects primary root growth *via* two successive processes: cell proliferation and cell elongation (Ruzicka et al., 2007; Street et al., 2015). Cell proliferation occurs at the RAM, which is composed of a quiescent center (QC), surrounding stem cells, and a group of mitotically active cells. The QC plays important roles in maintaining stem cell populations within the root meristem (Ortega-Martinez et al., 2007; Heyman et al., 2013). Accumulating evidence has revealed that ethylene induces irregular transverse cell divisions in the QC (Ortega-Martinez et al., 2007; Ni et al., 2014). Furthermore, ethylene inhibition of cell proliferation at the RAM is mainly achieved by restricting epidermal cell expansion (Street et al., 2015; Vaseva et al., 2018). Moreover, ethylene also inhibits the elongation of cells in the elongation zone. Ethylene stimulates auxin biosynthesis and basipetal auxin transport toward the elongation zone, where it activates a local auxin response leading to inhibition of cell elongation (Ruzicka et al., 2007). In summary, ethylene inhibits primary root growth by regulating cell proliferation in the RAM and cell elongation in the elongation zone.

In addition to ethylene, other plant hormones are also involved in regulating primary root growth, and accumulating data have shown that plant hormones interact with each other to regulate primary root growth (Thole et al., 2014; Pacifici et al., 2015; Van de Poel et al., 2015; Harkey et al., 2018). As a model plant of dicots and monocots, the results obtained in *Arabidopsis* and rice can be instructive for other species. In this current review, we summarize the current studies on the crosstalk of ethylene with other phytohormones during primary root development in *Arabidopsis* and rice, including auxin, ABA, GA, CKs, BRs, and JA, which contributes to better understanding of the role of ethylene in primary root development.

### Interaction of Ethylene and Auxin in Primary Root Growth

As an omnipotent regulator of root development, auxin inhibits the primary root elongation, and this effect is most probably mediated in crosstalk with ethylene (Hu et al., 2017; Qin and Huang, 2018). Auxin and ethylene act synergistically in the regulation of primary root elongation (Ruzicka et al., 2007; Swarup et al., 2007; Qin et al., 2017; Zemlyanskaya et al., 2018), and this regulation is mainly through the modulation of cell proliferation in the RAM and cell elongation in the elongation zone (Ruzicka et al., 2007; Street et al., 2015). The transcription of auxin biosynthesis and transport genes is regulated by ethylene, and the treatment of ethylene promotes auxin accumulation in the root tip, as revealed by indole-3-acetic acid (IAA) measurements and *DR5* reporter expression (Stepanova et al., 2005; Ruzicka et al., 2007; Qin et al., 2017; Mendez-Bravo et al., 2019). Moreover, disrupting auxin biosynthesis by L-kynurenine or yucasin [5-(4-chlorophenyl)-4H-1,2,4-triazole-3-thiol] impairs the ethylene effect on primary root growth (He et al., 2011; Qin et al., 2017). *Via* analysis of mutants that showed reduced sensitivity to ethylene in the primary roots, a set of *weak ethylene insensitive* (*wei*) mutants was isolated, such as *wei2/anthranilate synthase α1* (*asa1*), *wei7/anthranilate synthase β1* (*asb1*), and *wei8/*tryptophan aminotransferase of arabidopsis1 (taa1; Stepanova et al., 2005, 2008). *WEI2/ASA1* and *WEI7/ASB1* encode α and β subunits of anthranilate synthase, respectively, which is a rate-limiting enzyme involved in the biosynthesis of the auxin precursor tryptophan (Trp; Stepanova et al., 2005). *WEI8/TAA1* encodes key enzymes involved in the indole-3-pyruvic acid (IPyA) pathway, which is the most important pathway for producing auxin in plants (Stepanova et al., 2008; Mashiguchi et al., 2011). In addition to the defects of auxin biosynthesis, mutations affecting auxin transport, perception, or signaling also result in reduced sensitivity to ethylene (Hu et al., 2017; Merchante and Stepanova, 2017; Qin and Huang, 2018). Ethylene is one of the important players that regulate the auxin flow (Luschnig et al., 1998; Ruzicka et al., 2007; Stepanova et al., 2007; Swarup et al., 2007). Abnormal levels of ethylene can result in an imbalance in the auxin gradient, which in turn leads to a higher auxin content, and ultimately result in the inhibition of cell elongation and primary root growth. Mutations in influx carriers of the AUXIN1 (AUX1) family or efflux carriers of the PIN-FORMED (PIN) family lead to ethylene-resistance root phenotype (Luschnig et al., 1998; Ruzicka et al., 2007; Stepanova et al., 2007; Swarup et al., 2007). Loss of function of *AUXIN RESISTANT2* (*AXR2*) and *AXR3*, which are involved in the auxin signaling pathway, results in reduced ethylene sensitivity in primary root (Stepanova et al., 2007; Swarup et al., 2007). Mutants of auxin receptor *transport inhibitor response1* (*tir1*) show an ethylene-insensitive primary root growth (Figure 1; Alonso et al., 2003).

With an established method for screening ethylene-response mutants in rice, several mutants with defective ethylene response in primary root were identified (Ma et al., 2013). Among these mutants, some auxin biosynthesis- and signaling-related mutants, such as *maohuzi2* (*mhz2/sor1*) and *rice ethylene-insensitive7* (*rein7/yuc8*; Figure 1), have been identified (Qin et al., 2017; Chen et al., 2018). MHZ2, a RING finger E3 ubiquitin ligase, regulates ethylene response in primary roots by interacting with OsIAA26, an atypical Aux/IAA protein involved in the auxin signaling pathway (Chen et al., 2018). *REIN7* encodes an orthologue of *YUCCA8* (*YUC8*) and loss of *REIN7* function results in reduced auxin biosynthesis and ethylene insensitivity in primary roots (Qin et al., 2017). These studies suggest that ethylene-triggered inhibition of primary root elongation requires auxin biosynthesis, transport, and signaling, and the underlying mechanism is conserved in different species.

Emerging studies have shown that several transcription factors are involved in ethylene signaling or ethylene response, such as EIN3, the canonical transcription factor involved in the ethylene signaling pathway, regulating auxin biosynthesis by activating *YUC* transcripts (Liu et al., 2016; Qin et al., 2017). ETHYLENE RESPONSE FACTOR1 (ERF1), a direct target of EIN3, is responsive to ethylene and directly activates *ASA1*. ASA1 encodes a rate-limiting enzyme in Trp biosynthesis where auxin is derived. The activation of *ASA1* promotes auxin biosynthesis, results in the increased auxin accumulation, and thus inhibits primary root elongation (Solano et al., 1998; Mao et al., 2016). HOMEOBOX PROTEIN52 (HB52), which acts downstream of EIN3, binds to the promoters of *PIN2*, *WAVY ROOT GROWTH1* (*WAG1*), and *WAG2* to increase their expression. WAG1 and WAG2 phosphorylate PIN2, thus modulating auxin transport (Figure 1; Miao et al., 2018). These transcription factors act as nodes in the crosstalk between ethylene and auxin in primary root growth, implying that ethylene regulates primary root growth by modulating auxin biosynthesis, transport, and signaling.

### Coordination of Ethylene and Abscisic Acid in Primary Root Growth

ABA regulates many aspects of plant growth and development, including primary root elongation (Luo et al., 2014; Li et al., 2017; Sun et al., 2018). Studies have shown that ABA has biphasic effects on primary root growth, depending on its concentration, environmental conditions, developmental context, genotypes, and plant species. Typically, low concentrations of ABA stimulate primary root growth, whereas high concentrations inhibit it (Rowe et al., 2016; Li et al., 2017).

Increasing numbers of studies in *Arabidopsis* have suggested that ABA inhibits primary root growth mainly acting on cortical cells in the elongation zone, and this process requires the ethylene signaling pathway (Beaudoin et al., 2000; Ghassemian et al., 2000; Dietrich et al., 2017). The ethylene signaling mutant *ethylene response1* (*etr1-1*) and *ein2* showed resistance to ABA-inhibited primary root growth, whereas the ABA-resistant mutant *ABA-insensitive1* (*abi1-1*) and the ABA-deficient mutant *aba2* exhibited a normal ethylene response in the roots (Beaudoin et al., 2000; Ghassemian et al., 2000; Thole et al., 2014). Moreover, disruption of ABA biosynthesis or signaling in the *ein2*, *ein3,* or *ctr1* mutant background by introducing the *aba2* or *abi1* mutation did not alter the ethylene response phenotypes of the respective ethylene mutants (Beaudoin et al., 2000; Cheng et al., 2009). The above results suggest that ABA-inhibited primary root growth requires a functional ethylene signaling pathway but that ethylene-inhibited root growth is ABA-independent. Recent studies have indicated that ABA inhibits primary root growth by increasing ethylene biosynthesis (Luo et al., 2014). ABA activates two calcium-dependent protein kinases, CPK4 and CPK11, which phosphorylate the C-terminus of ACS6 and increase the stability of ACS6 in ethylene biosynthesis, thus promoting the biosynthesis of ethylene (Figure 1). Disruption of ethylene biosynthesis by AVG relieves the inhibitory effect of ABA on primary root growth (Luo et al., 2014; Li et al., 2017). In addition, a protein phosphatase type 2C, ABI1, a negative regulator of ABA signaling, negatively regulates ethylene biosynthesis by counteracting the phosphorylation of ACS2/ACS6 mediated by MITOGENACTIVATED PROTEIN KINASE 6 (MAPK6; Figure 1; Ludwikow et al., 2014). Together, these results suggest that ABA treatment induces ethylene biosynthesis, thus leading to the inhibition of primary root growth, and that ABA responses require normal ethylene signaling.

ABA and ethylene crosstalk in rice have been revealed by two ethylene-response mutants: *mhz4* and *mhz5* (Ma et al., 2014; Yin et al., 2015). MHZ4 is a homologue of *Arabidopsis* ABA4, which is involved in ABA biosynthesis. *MHZ5* encodes a carotenoid isomerase, which is essential for ABA biosynthesis in etiolated rice shoots and roots. Mutations in *MHZ4* and *MHZ5* reduce the ethylene response in roots but enhance the ethylene response in the coleoptiles of etiolated seedlings. Overexpression of *MHZ4/5* results in enhanced ethylene sensitivity in roots and reduced ethylene sensitivity in coleoptiles. Genetic studies have revealed that the MHZ4/5-mediated ABA pathway in rice acts downstream of ethylene signaling to inhibit root growth (Figure 1), which is different from that in *Arabidopsis*, where ABA inhibits root growth through promoting ethylene biosynthesis, suggesting that different mechanisms have evolved in these two species. In addition, the MHZ4/5-mediated ABA pathway in rice acts upstream of ethylene signaling to control coleoptile growth (Ma et al., 2014; Yin et al., 2015), suggesting that the interaction between ethylene and ABA is distinctive in different tissues.

### Integration of Ethylene and Gibberellins in Primary Root Growth

GAs also play an important role in primary root growth. Mutation in GA biosynthesis genes or disruption of GA biosynthesis by paclobutrazol, an inhibitor of GA biosynthesis, substantially reduces the rate of cell proliferation in the *Arabidopsis* root meristem (Achard et al., 2009; Ubeda-Tomas et al., 2009; Lee et al., 2012). GAs have been proposed to promote root growth in *Arabidopsis* by increasing the elongation of both dividing and post-mitotic endodermal cells, thereby indirectly controlling the division and elongation of other types of root cells and the overall root meristem size (Ubeda-Tomas et al., 2009).

GAs are perceived in the cell through a simple pathway that is regulated by a family of proteins known as DELLA proteins, a subfamily of the GRAS family of putative transcriptional regulators (Richards et al., 2001; Daviere and Achard, 2013). In *Arabidopsis*, the DELLA family comprises GA-INSENSITIVE (GAI), REPRESSOR OF GA (RGA), RGA-LIKE1 (RGL1), RGL2, and RGL3 (Peng et al., 1997; Silverstone et al., 1998). GAs promote the degradation of DELLA proteins. However, ethylene treatment delays the GA-induced diminishing of green fluorescent protein (GFP)-RGA fusion constructs from root cell nuclei *via* a CTR1-dependent signaling pathway (Figure 1). Moreover, mutations in GAI and RGA reduce the sensitivity to ethylene in primary root growth, and exogenous GA treatment substantially releases the inhibition by ethylene of primary root growth (Achard et al., 2003), suggesting that ethylene and GA act antagonistically in primary root growth.

In rice, GA participates in the modulation of cell elongation and proliferation in the root meristem, GA deficiency leads to short primary roots (Li et al., 2015), and exogenous applications of GA can increase primary root growth and ethylene production (Lee and Yoon, 2018). *Via* an analysis of the ethylene-responsive genes in the roots of *mhz7/osein2* and *mhz6/oseil1* compared with the wild type by RNA sequencing, some genes are involved in GA biosynthesis and the catabolic pathway has been identified (Yang et al., 2015). Considering that ethylene inhibits primary root growth, while GA promotes it, ethylene may reduce endogenous GA contents by the transcriptional regulation of GA biosynthesis and catabolic genes in primary roots.

### Interactions Between Ethylene, Cytokinins, Jasmonic Acid, and Brassinosteroids in Primary Root Growth

CKs are widely considered to inhibit primary root growth in *Arabidopsis* (Cary et al., 1995; Riefler et al., 2006; Ioio et al., 2012). Accumulating studies showed that CKs regulate primary root growth by interacting with ethylene, and this interaction mainly occurs in cell proliferation in the RAM (Street et al., 2015; Van de Poel et al., 2015; Liu et al., 2017). In *Arabidopsis*, treatment of etiolated seedlings with CKs produces a phenotype similar to ethylene (Cary et al., 1995), suggesting that CKs and ethylene may act synergistically to regulate specific growth and developmental processes. Further research shows that CK treatment increases the stability of the ethylene biosynthesis protein ACS5, leading to increased ethylene production (Figure 1; Chae et al., 2003; Hansen et al., 2009). Proteome analysis in *Arabidopsis* roots reveals that CKs upregulate the majority of proteins in the ethylene biosynthesis pathway (Zdarska et al., 2013). These results suggest that CKs inhibit primary root growth through enhancing ethylene biosynthesis. However, Kushwah et al. (2011) reported that CKs can induce primary root elongation, and this response is mediated by the ethylene signaling pathway through the ethylene receptor ETHYLENE RESISTANT1 (ETR1) and its downstream signaling element EIN2, suggesting that the ethylene signaling pathway is required for CK-induced primary root growth response. In rice, recent research suggests that exogenous CKs upregulate the transcription of ethylene biosynthesis genes in primary roots, resulting in increased ethylene biosynthesis and inhibition of primary root growth (Zou et al., 2018).

The function of JA in plant resilience to many environmental challenges has been well studied, and its role in root growth has also been reported (Wasternack and Hause, 2013; Huang et al., 2017; Guo et al., 2018). Exogenous application of JA inhibits various aspects of seedling growth, including primary root growth (Wasternack and Hause, 2013; Huang et al., 2017). In response to the JA signal, the F-box protein CORONATINE INSENSITIVE1 (COI1) recruits JASMONATE ZIM-domain (JAZ) repressors for ubiquitination and degradation, thereby relieving the repression of transcription factors and enabling the expression of JA-responsive genes and JA responses (Thines et al., 2007; Katsir et al., 2008; Fernandez-Calvo et al., 2011; Qi et al., 2015). Complicated modes of interaction between JA and ethylene have been investigated in different processes. JA enhances aluminum-induced primary root growth inhibition, and this process is controlled by ethylene. Aluminum-induced upregulation of pCOI1:COI1-VENUS in the root apex transition zone was significantly repressed in the *ein3eil1* double mutant, suggesting that JA acts downstream of ethylene in aluminum-induced primary root growth inhibition (Yang et al., 2017). In addition, JA and ethylene act synergistically to regulate root hair development, through direct interaction of JASMONATE ZIM-DOMAIN (JAZ) proteins, which are degraded after JA treatment, with EIN3/EIL1 to attenuate its transcriptional activity (Zhu et al., 2006, 2011). The above studies suggest that different mechanisms are present in different tissues, and additional studies are needed to elucidate the involvement of JA-ethylene crosstalk in primary root growth.

BRs, a class of plant-specific steroid hormones, play important roles in regulating primary root growth. Low concentrations of BRs can induce root growth, while high concentrations inhibit root growth (Clouse et al., 1996; Mussig et al., 2003; Lv et al., 2018). Mutations in BR biosynthesis or signaling pathway exhibit reduced cell proliferation in the RAM and decreased cell length in the mature zone, thus leading to shortened roots (Li et al., 1996; Mussig et al., 2003; Lv et al., 2018). BRs, perceived by the BRASSINOSTEROID INSENSITIVE1 (BRI1) receptor, activate the transcription factors BRI1-EMS-SUPPRESSOR1 (BES1) and BRASSINOSTEROID RESISTANT1 (BZR1), which in turn induce the BR response (Yin et al., 2002; Yu et al., 2011; Oh et al., 2012). Several studies have shown that BRs regulate primary root growth through modulating ethylene biosynthesis, namely, low levels of BRs inhibit ethylene biosynthesis by activating BZR1 and BES1 to repress the expression of *ACSs* (Lv et al., 2018). Although high levels of BRs induce ethylene biosynthesis by increasing the stability of ACSs (Figure 1), these dual effects on ethylene lead to biphasic effects of BRs on primary root growth (Lim et al., 2002; Hansen et al., 2009; Lv et al., 2018).

## Conclusions and Perspectives

During the past few years, multiple studies have provided molecular connections between ethylene and primary root growth. In this review, we integrated recent data showing the molecular details of the interactions between ethylene and other plant hormones in the regulation of primary root development (Figure 1). In particular, ethylene regulates primary root growth through modulating auxin biosynthesis, transport, and signaling, and this regulatory mechanism is conserved in *Arabidopsis* and rice. ABA and CKs inhibit primary root growth in *Arabidopsis* by affecting the posttranscriptional regulation of ACS, which results in increased ethylene synthesis. However, in rice, ethylene inhibition of primary root growth requires ABA function, and this mode of ethylene-ABA interaction is fundamentally different from that in *Arabidopsis*, suggesting that different mechanisms exist in different species. GA and ethylene antagonistically regulate the stability of DELLA proteins, which act as growth repressors. BRs have biphasic effects on primary root growth, namely, low concentrations of BRs inhibit ethylene biosynthesis by activating BZR1 and BES1 to repress the expression of *ACSs*, whereas high levels of BRs induce ethylene biosynthesis through increasing the stability of ACSs. Based on the above results, a general interaction model where ethylene plays a central role is presented (Figure 1). Thus, this review provides an overview on the crosstalk of ethylene and other hormones in primary root growth, increasing our understanding of the regulation of ethylene in primary root growth.

Primary root growth begins during embryo development and is easily affected by adverse soil conditions. Plant hormones act as all-encompassing regulators of normal root growth and mediate root morphological responses to abiotic stresses (De Smet et al., 2015). Further investigations should focus on how plants perceive external changes and translate cues into adaptive responses by modulating endogenous hormone crosstalk dynamics. Moreover, there is a complex regulatory network among phytohormones, and different regulatory mechanisms exist in different tissues, at different developmental stages, and in different species (Luo et al., 2014; Ma et al., 2014; Yin et al., 2015; Abts et al., 2017). Thus, researchers should try to understand hormone crosstalk in a multidimensional space, and the development of effective hormone detection methods and computational models will greatly promote research on hormone crosstalk.

With the advancement of technology, high-throughput technology provides an opportunity to track complex regulatory pathways during primary root growth for detecting the molecular mechanisms that govern hormone crosstalk and the nodes of interactions between different hormone pathways (Hung and Weng, 2017). However, caution and further validation are required when using those data. In addition, it is also interesting to clarify the regulatory network of ethylene and other hormones in multiple cell types. Single-cell transcriptome sequencing can provide us with an overview of ethylene and other hormone actions in specific cells (Ziegenhain et al., 2017; Denyer et al., 2019), and cell type-specific promoters allow us to study the function of a desired protein in particular cell types and to reveal the main sites of ethylene and other hormone actions (Vaseva et al., 2016).

## Author Contributions

All the authors discussed and created the review’s outline. HQ wrote the manuscript. RH edited the manuscript.

### Conflict of Interest Statement

The authors declare that the research was conducted in the absence of any commercial or financial relationships that could be construed as a potential conflict of interest.
